# Electrochemical Detection of Fluoroquinolone Antibiotics in Milk Using a Magneto Immunosensor

**DOI:** 10.3390/s140915965

**Published:** 2014-08-28

**Authors:** Daniel G. Pinacho, Francisco Sánchez-Baeza, María-Isabel Pividori, María-Pilar Marco

**Affiliations:** 1 Nanobiotechnology for Diagnostics Group (Nb4D), IQAC-CSIC and CIBER de Bioingeniería, Biotemateriales y Nanomedicina, c/ Jordi Girona 18-26, 08034 Barcelona, Spain; E-Mails: daniel.gonzalez@iqac.csic.es (D.G.P.); francisco.sanchez@iqac.csic.es (F.S.-B.); 2 Sensors & Biosensors Group, Autonomous University of Barcelona (UAB), 08013 Bellaterra, Barcelona, Spain; E-Mail: Isabel.Pividori@uab.cat

**Keywords:** electrochemical immunosensor, fluoroquinolone antibiotics, antimicrobial residues, magnetic beads, food safety, milk

## Abstract

An amperometric magneto-immunosensor (AMIS) for the detection of residues of fluoroquinolone antibiotics in milk samples is described for the first time. The immunosensor presented combines magnetic beads biomodified with an antibody with a broad recognition profile of fluoroquinolones, a haptenized enzyme and a magnetic graphite–epoxy composite (m-GEC) electrode. After the immunochemical reaction with specific enzyme tracer, the antibody biomodified magnetic beads are easily captured by an electrode made of graphite-epoxy composite containing a magnet, which also acts as transducer for the electrochemical detection. In spite of the complexity of milk, the use of magnetic beads allows elimination of potential interferences caused by the matrix components; hence the AMIS could perform quantitative measurements, directly in these samples, without any additional sample cleanup or extraction step. The immunosensor is able to detect up to seven different fluoroquinolones far below the MRLs defined by the UE for milk; for example ciprofloxacin is detected directly in milk with an IC_50_ of 0.74 μg/L and a LOD of 0.009 μg/L. This strategy offers great promise for rapid, simple, cost-effective, and on-site analysis fluoroquinolones in complex samples.

## 1. Introduction

In modern intensive farming systems, the introduction of veterinary pharmaceuticals, pesticides and complex animal feeds has been critical to enhance the productivity by increasing the density of animals in the farms. In this sense, antibiotics are used for therapy, prevention and control of diseases, as well as growth promoters. The use of antibiotics is a determinant factor in the selection of resistance in bacteria and the dosage of antimicrobials is closely related to the rate of antimicrobial resistance developed in bacteria [1–3]. Development of resistance in zoonotic bacteria constitutes a public health concern, primarily due to the increased risk of treatments failure of human diseases. Food animals and foodstuffs are traded worldwide, thus, the occurrence of antimicrobial resistance is a worldwide problem.

Fluoroquinolones (FQs) are synthetic antibiotics with a broad spectrum of antimicrobial activity that have been extensively used in veterinary medicine to treat or prevent bacterial infections in food-producing animals, aquaculture, and also pets. Several FQs are available for therapy of animals in many countries; however, the usage of these antibiotics differs greatly as regards animal species, indications, label indications, and geographic spread [4]. Because of the concerns regarding drug residues entering the food chain and contaminating environment, and consequently contributing to bacterial resistance development, maximum residue levels (MRLs) for the detection of FQ in several matrices from diverse animal species have been established by the European Commission (Council regulation 2377/90/EC [5] and council directive 96/23/EC [6]), as well as the requirements of the analytical methods that public health control and veterinary laboratories should use for this purpose (Commission decision 2002/657/EC [7]). In order to fulfill these requirements, and with the aim to protect the public health, official laboratories should be able to efficiently process a high number of samples at a low cost. The most employed analytical methods for the determination of FQs antibiotics in foods of animal origin are high-performance liquid chromatography (HPLC) coupled to fluorescence, mass spectrometric and ultraviolet detection [8–10]. These methods are considered as “gold standard” and they are used as routine in many laboratories. However, the expensive equipment required and the need for sample treatment limits their implementation in some settings. Although they are very suitable for confirmation, their use for screening purposes of large numbers of test samples become expensive. As a consequence, development of rapid, inexpensive and sensitive high sample throughput or on-site analytical strategies would be desirable.

Antibodies based assays can provide the necessary reliability, low cost of the analysis/sample, ease of use, selectivity, and detectability to analyze small organic molecules. Moreover, the use of magnetic beads improves performance of the immunochemical reaction, due to an increase in the surface area, as well as achieving faster assay kinetics. Despite this increased surface area, matrix effect can be minimized due to their properties, as well as the improved washing and separation steps [11,12]. On the other hand, electrochemical sensors have several advantages, such as their simplicity, portability, and low cost, giving them attractive sensing characteristics for their use as alternative analytical methods, with accurate and fast responses without sample pre-treatment, opening up the possibility of direct on-site analysis with intuitive devices [13]. In this sense, electrochemical immunosensors combine the high sensitivity of the electrochemical transducers with the high selectivity of antibodies, thus, becoming powerful analytical tools for the detection of antibiotic residues [14,15]. Recently, Conzuelo *et al.* have been developed an immunosensor for the detection of tetracyclines antibiotics in milk using magnetic beads. In this case, they used a homemade magnet holder block to capture the magnetic beads onto a disposable commercial screen-printed electrodes, achieving good detectability in milk diluted two times in buffer [16].

In this paper an amperometric magneto-immunosensor (AMIS) based on the use of specific antibody biomodified magnetic beads, which can be captured by a magnetic graphite–epoxy composite (m-GEC) electrode is presented as a rapid, sensitive, simple, inexpensive, and user-friendly analytical method for the detection of fluoroquinolone antibiotics in milk. The device uses and enzyme tracer to generate electrochemical species and shows a broad selectivity profile for this antibiotic family. Fluoroquinolone residues can be directly detected in milk samples, without the need of previous cleanup or purification steps.

## 2. Experimental Section

### 2.1. Materials and Instruments

Amperometric measurements were performed with a VersaSTAT 3 potentiostat (Princeton Applied Research, TN, USA). A three-electrode setup was used comprising a XM120 8 mm platinum plate auxiliary electrode (Radiometer Analytical SAS, France), double junction Ag/AgCl reference electrode (Orion 900200) with 0.1 M KCl as the external reference solution and a working electrode (magnetic graphite–epoxy composite (m-GEC)). The m-GEC was prepared using graphite powder with a particle size of 50 μm (BDH, UK) and Epo-Tek H77 (epoxy resin from Epoxy Technology, USA). Tosylactivated magnetic beads (Dynabeads® M-280 Tosylactivated) were purchased from Invitrogen Dynal AS (Oslo, Norway). Polystyrene microtiter plates were purchased from Nunc (Maxisorb, Roskilde, DK). Washing steps in ELISA were carried out using a SLT 96PW microplate washer (SLT Labinstruments GmbH, Salzburg, Austria). The electrochemical measurements performed were analyzed using VersaStudio software (Princeton Applied Research, TN, USA). To perform the optical measurements a SpectramaxPlus microplate reader (Molecular Devices, Sunnyvale, CA, USA) was used. The calibration curves were fitted to a four-parameter logistic equation using the Graph Prism software (GraphPad Software, San Diego, CA, USA). The magnetic separation during the washing steps was performed using a magnetic separator Dynal MPC-S (Dynal Biotech ASA, Norway) or 96-Well Plate Separation Rack (Cortex Biochem, CA, USA). The pH and conductivity of all buffers and solutions were measured with a pH meter pH 540 GLP and a conductimeter LF 340, respectively (WTW, Weilheim, Germany).

### 2.2. Chemicals and Immunochemicals

The immunoreagents used in this study (Ab171 and 11-BSA), as well as the fluoroquinolone hapten 11 were produced as previously described [17]. Ab171 was used as a pure IgG fraction of the corresponding antisera (As171) and it was obtained by ammonium sulfate precipitation [18] followed by affinity chromatography purification (HiTrap Protein A HP, 1 mL, Amersham Biosciences, UK). Horseradish peroxidase (HRP) and bovine serum albumin (BSA) were purchased from Sigma (St. Louis, MI, USA). Ciprofloxacin was kindly provided by UQUIFA S.A (Lliça de Vall, Spain). The other fluoroquinolones used for crossreactivity studies were acquired from Aldrich Chemical Co. (Milwaukee, WI, USA). All the other chemical reagents used for the preparation of the bioconjugates, the biomodification of magnetic beads and the purification of the antibodies were of the highest available grade and were purchased from Fluka-Sigma-Aldrich (St. Louis, MO, USA) or Merck (Darmstadt, Germany). Stock solutions of different fluoroquinolones (0.01 M) were prepared in 0.05 M NaOH solution and stored at 4 °C for one month.

### 2.3. Buffers and Solutions

Phosphate-buffered saline (PBS) is 0.01 M phosphate buffer on a 0.8% saline solution, and the pH is 7.5. PBST is PBS with 0.05% Tween 20. PBST_Ca_ is PBST with 1 mM calcium chloride. Coating buffer is 0.05 M carbonate-bicarbonate buffer, pH 9.6. Citrate buffer is a 0.04 M solution of sodium citrate, pH 5.5. The substrate solution for optical measurements contains 0.01% TMB (3,3′,5,5′-tetramethylbenzidine) and 0.004% H_2_O_2_ in citrate buffer. For electrochemical measurements, PBS_E_ (0.1 M phosphate buffer, with 0.1 M KCl, pH 7) was used. During biomodification of the magnetic beads, PBS containing 0.1% (w/v) BSA (PBS_BSA_), and 0.2 M tris(hydroxymethyl)aminomethane (Tris), pH 8.5 containing 0.1% (w/v) BSA (Tris_BSA_) were used. All buffer solutions were prepared with ultrapure water (UPW, D11971 Barnstead).

### 2.4. Construction of Magneto Graphite–Epoxy Composite (m-GEC) Electrodes

The magnetic graphite–epoxy composite (m-GEC) electrodes were constructed following the protocol previously reported for electrochemical immunosensing [19–21]. Briefly, epoxy resin and graphite powder were hand-mixed in a 1:4 (w/w) ratio. Then, a cylindrical PVC sleeve body (6 mm i.d.), with a copper plate as electrical contact, was filled with the resulting paste and a small neodymium magnet (3 mm i.d.) was placed into the center of this electrode. After that, tightly packed the GEC paste in the electrode body gap, the m-GEC electrodes were cured at 40 °C for 1 week to obtain a rigid composite. Before each measurement, the electrode surface was renewed by a simple polishing procedure, wetted with UPW, and then thoroughly smoothed with abrasive paper.

### 2.5. Preparation of the Enzyme Labeled Immunoreagents

HRP was covalently coupled to Ab171 and fluoroquinolone hapten 11 through a two steps procedure, previously described by Abuknesha [22], with modifications.

Step 1: Activation of HRP. A solution of HRP (9 mg, 0.225 μmol) in coating buffer (1.8 mL) was added to cyanuric chloride (900 μg, 4.9 μmol) and the mixture was stirred during two hours at room temperature (RT). Afterwards, the mixture was dialyzed (4 × 0.5 mM PBS and 1 × UPW) and finally freeze-dried to obtain 8.02 mg of HRP_CC_ (89% of yield).
Step 2: Bioconjugation. A solution of Ab171 (1.8 mg, 0.012 μmol) in coating buffer (250 μL) and a solution of hapten 11 (0.6 mg, 1.95 μmol) in DMF (60 μL) were added to a two solutions of HRP_CC_ (3 mg, 0.07 μmol) in coating buffer (500 and 690 μL respectively) and the mixtures were stirred for 4 h at 37 °C. After that, the mixture of the enzyme labeled antibody was purified by 40% (NH_4_)_2_SO_4_ precipitation [18] and the precipitate was restored with PBS, pH 7.5. Finally, the solutions of enzyme labeled antibody and enzyme tracer were dialyzed (4 × 0.5 mM PBS and 1 × UPW) and freeze-dried to obtain 1.23 mg of Ab171-HRP (53% of yield) and 2.11 mg of 11-HRP (70% of yield). All immunoreagents were stored at −80 °C and unless otherwise indicated, working aliquots were stored at 4 °C in PBS at 1 mg/mL.

### 2.6. Preparation of the Biomodified Magnetic Beads

The 11-BSA bioconjugate and the previously purified Ab171 were covalently coupled to MB-Tosyl magnetic beads to obtain the corresponding modified magnetic beads 11-BSA-MB and Ab171-MB. According to the manufacturer specifications, covalently biomodified magnetic beads can be stored at 4 °C for a minimum of three months without a loss of activity. A volume of 350 μL (30 mg/mL, 0.1–0.2 mmol/g) of tosyl-modified magnetic beads (10.5 mg), were washed twice with 1 mL of coating buffer, avoiding foaming. After that, the magnetic beads were suspended in coating buffer (850 μL) and a solution of either 11-BSA or Ab171 (1 mg/mL, 150 μL) was then added. The magnetic beads were incubated 48 h at RT with a slow orbital rotation in order to avoid bead sedimentation. The modified beads were then washed twice in PBS_BSA_ for 5 min at 4 °C and with Tris_BSA_ for 24 h at RT with slow orbital rotation and washed again with PBS_BSA_ for 5 min at 4 °C. Finally, the magnetic beads were resuspended in PBS_BSA_ to reach a 15 mg/mL stock solution and were stored at 4 °C. The yield of the coupling was evaluated by means of a UV test (δ = 280 nm), in which the protein concentration in the supernatant was analyzed before and after the bioreaction. The biomodification yield, calculated as described above, was 82.5 ± 1% for 11-BSA-MB and 92 ± 2% for Ab171-MB.

### 2.7. ELISA and m(magneto)-ELISA Protocol

#### ELISA

Two different competitive formats were assessed in this study, coating antigen format (CA_f_) comprising the immunoreagents Ab171-HRP and 11-BSA and enzyme tracer format (ET_f_) comprising the reagents 11-HRP and As171.

Microtiter plates were coated with 11-BSA (0.5 μg/mL, 100 μL/well) and As171 (8000 times diluted, 100 μL/well) for CA_f_ and ET_f_, respectively, both of them in coating buffer at RT for 4 h and covered with adhesive plate sealers. Then, the plates were washed with PBST (four times, 300 μL/well) and ciprofloxacin standard solutions (from 0.01 nM to 10,000 nM, in PBST_Ca_, 50 μL/well) were added to the microtiter plates, followed by the addition of Ab171-HRP (0.25 μg/mL in PBST_Ca_, 50 μL/well) and 11-HRP (0.25 μg/mL in PBST_Ca_, 50 μL/well) for CA_f_ and ET_f_ respectively. After 30 min, the plates were washed again, and the substrate solution was added (100 μL/well). Color development was stopped after 30 min at RT with 2 M H_2_SO_4_ (50 μL/well), and the absorbances were read at 450 nm.

#### m-ELISA

Two different competitive formats were assessed in this study, magneto coating antigen format (m-CA_f_) comprising the immunoreagents Ab171-HRP and 11-BSA-MB and magneto enzyme tracer format (m-ET_f_) comprising the reagents 11-HRP and Ab171-MB.

Ciprofloxacin standard solutions in PBST_Ca_ (50 μL, from 0.01 to 10,000 nM) were mixed with a suspension of the biomodified magnetic beads (50 μL, 0.3 mg/mL of 11-BSA-MB for m-CA_f_ and 0.125 mg/mL of Ab171-MB for m-ET_f_) followed by the addition of the enzyme labeled immunoreagent solutions (50 μL, 0.5 μg/mL of Ab171-HRP for m-CA_f_ and 0.25 μg/mL of 11-HRP for m-ET_f_). The competitive immunological reaction was allowed to proceed for 30 min at RT vigorously shaking. The magnetic beads were then washed with PBST (150 μL, three times) and the substrate solution (100 μL) was added and incubated again for 30 min at RT. The enzymatic reaction was stopped by adding 2 M H_2_SO_4_ (50 μL). Finally, the supernatants were removed from the magnetic beads and then added to a different plate for measuring the absorbance at 450 nm.

In both cases (ELISA and m-ELISA), the standard curve was fitted to a four-parameter logistic equation according to the formula y = (A − B)/[1 + (X/C) D] + B, where A is the maximal signal, B the minimum signal, C the concentration producing 50% of the maximal signal, and D is the slope at the inflection point of the sigmoid curve. LOD values were obtained as 90% of A value, the maximal signal.

### 2.8. Specificity Studies

Stock solutions of different fluoroquinolone antibiotics were prepared in 0.05 M NaOH at a concentration of 0.01 M. Standard solutions were prepared for each analyte in PBST_Ca_ (from 0.01 nM to 10,000 nM) and measured using the ELISA and m-ELISA, as described above. For all fluoroquinolones it was possible to build standard curves that fitted to the four-parameter equation mentioned above. The cross-reactivity values were calculated according to the equation: (IC_50_ Ciprofloxacin/IC_50_ tested compounds) × 100.

### 2.9. Amperometric Magneto Immunosensor (AMIS) Procedure

**Figure 1.**
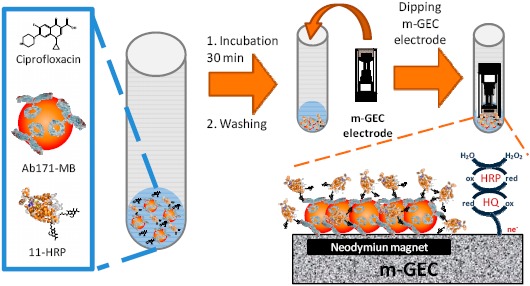
Scheme of the AMIS procedure for fluoroquinolones. Firstly, ciprofloxacin standards, Ab171-MB suspension and 11-HRP solution were mixed and incubated for 30 min with gentle stirring. After three washing steps using PBST buffer, performed with the aid of a magnetic rack, the modified magnetic beads are captured by the m-GEC. Then, the electrode is transported to the electrochemical cell using a three-electrode setup for the amperometric measurement.

For electrochemical readout (AMIS), only the magneto enzyme tracer format (m-ETf), comprising the reagents 11-HRP and Ab171-MB, was assessed. A schematic representation of the whole procedure is shown in Figure 1. All the steps were performed in 5 mL polypropylene tubes, and all the referred quantities in this protocol are the amount added per tube. After the incubation or washing steps, the magnetic beads were separated from the supernatant using a magnetic separation rack until the beads migrated to the sides of the tube and the liquid was clear (approximately 1 min).

Ciprofloxacin standard solutions (50 μL, from 0.01 to 10,000 nM in PBST_Ca_) were placed in the polypropylene tubes and mixed with the Ab171-MB suspension (50 μL, 0.25 mg/mL in PBST_Ca_) and the solution of 11-HRP (50 μL, 0.5 μg/mL in PBST_Ca_), in this specific order, and incubated for 30 min at RT with gentle stirring. Afterwards, the magnetic beads were washed with PBST (3 × 300 μL), resuspended in PBST (160 μL) and captured by dipping the m-GEC electrode into the solution in the tubes just before the amperometric measurement in order to prevent undesired desorption of the protein complexes (see Figure 1). The magnetic beads-modified m-GEC was used as a working electrode to determine the amperometric signal by dipping the three-electrode setup in 20 mL of PBS_E_. The electrochemical signal was recorded after the addition of the mediator (hydroquinone, HQ; 1.81 mM) and the enzyme substrate (hydrogen peroxide, 1.11 mM). The response was determined by polarizing the working electrode surface (m-GEC) at −0.150 V. As well as for ELISA and m-ELISA, the standard curve was fitted to a four-parameter logistic equation according to the formula (see above).

### 2.10. Milk Matrix Effect

Bovine whole milk samples testing free of fluoroquinolones were provided by the Agencia Española para la Seguridad Alimentaria (AESA; Spanish Agency for Food Security). Different dilutions of whole bovine milk were employed for generate ciprofloxacin standard curves. The curves obtained using ELISA, m-ELISA and AMIS procedures were compared with the curve prepared in PBST_Ca_ buffer in order to evaluate the matrix effect.

## 3. Results and Discussion

The AIMS presented here, is based on the use of an enzyme tracer, magnetic beads modified with antifluoroquinolone antibodies, and graphite–epoxy composite electrodes containing a magnet inside (m-GEC electrodes). The samples or standards of fluoroquinolones are mixed with the immunoreagents listed above, and after incubation, the immunocomplexes linked to the magnetic beads are separated from the free fraction of the enzyme tracer with the support of a magnetic rack and later on captured with the m-GEC (see Figure 1). The amperometric signal is determined by using the magnetic beads-modified m-GEC electrode as a working electrode in a three-electrode setup, recording the current intensity produced by the enzymatic reaction catalyzed by the HRP of the immunocomplex. The amperometric signal is related to the amount of enzyme tracer bound to the magnetic beads captured by the electrode that is inversely proportional to the amount of analyte present in the sample. The enzyme oxidizes the substrate (H_2_O_2_ to H_2_O) at the same time that hydroquinone (HQ) is oxidized to quinone (Q), and subsequently reduced again by the electrode applied potential (see Figure 1).

### 3.1. Preparation and Characterization of Enzyme Labeled Immunoreagents

Recently, our group has developed generic antibodies against fluoroquinolones and an indirect competitive ELISA assay able to detect, at least, the most important fluoroquinolones used in the veterinary field with values of IC_50_ and LOD below the MRLs established by the UE for several tissues [17]. This ELISA format comprises a competition step between the coating antigen and the analyte for the antibody, and also implies a second step using a secondary anti-antibody labeled with an enzyme for the generation of the final signal. With the aim to avoid this second step, halving the analysis time, two new enzyme labeled immunoreagents were prepared and evaluated by ELISA. The first one was prepared by direct coupling of HRP to the antifluoroquinolone antibody (Ab171) and the second one by coupling the same enzyme to the fluoroquinolone hapten (11) that showed the best performance in the ELISA previously developed.

**Figure 2.**
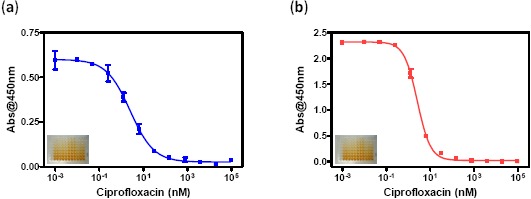
ELISA calibration curves of the different ELISA formats using the immunoreagents produced (**a**) CA_f_ in blue; (**b**) TE_f_ in red. Each point was the average of at least three-well replicates, and the assays were run on two different days.

For the preparation of the labeled specific antibody, two methodologies were employed. In a first approach the sugars of the antibodies were oxidized by sodium periodate, in order to obtain aldehyde functionalities able to react with the amines of the HRP [23]. In this case the enzyme labelled antibody obtained showed a high degree of non-specific absorption when it was characterized by ELISA assay (data don't show), for this reason this strategy was discarded. In a second approach, cyanuric chloride was employed as crosslinker using the different reactivity of the chlorine atoms [22]. The most reactive chlorine moiety of the linker reacted, on a first step, with the amines of the HRP at RT. After purification of the activated HRP, the second reactive chlorine of the crosslinker was reacted with the amines of the lys residues of the antibody at 37 °C. In this case an enzyme labeled antibody (Ab171-HRP) showing very good performance in ELISA assay was obtained (see Figure 2 and Table 1). Thus, in the same way, an enzyme tracer (11-HRP) was prepared by the reaction of the amine group of the fluoroquinolone hapten 11 with the chlorine atom of the activated HRP a 37 °C. Both bioconjugates were tested by ELISA using distinct formats (CA_f_, coating antigen and ET_f_, enzyme tracer formats) The CA_f_ ELISA assay, performed using plates coated with the competitor 11-BSA and Ab171-HRP in solution (see experimental Section 2.7), showed a very good detectability for ciprofloxacin with an IC_50_ of 0.84 μg/L and a LOD of 0.06 μg/L. The enzyme tracer was evaluated using an ET_f_ ELISA, using plates coated with the antifluoroquinolones antisera (As171) without previous purification (see experimental Section 2.7). Again, a very good detectability was accomplished (see Figure 2 and Table 1), reaching an IC_50_ of 0.86 μg/L and a LOD of 0.09 μg/L for ciprofloxacin.

The yield of the bioconjugation for 11-HRP (63%) was higher than for the Ab171-HRP (47%), maybe due to a minor purification steps involved during the process. Both ELISA formats show a very close detectability in terms of IC_50_ and LOD for ciprofloxacin, and similar to the previous ELISA developed (IC_50_ = 0.35 μg/L, LOD = 0.04 μg/L), demonstrating that there was no loss of activity after labeling of the specific antibody. On the other hand, the similarity found between both formats indicates that the assay is not dependent on the format.

### 3.2. Preparation and Characterization of Biomodified Magnetic Beads

**Figure 3.**
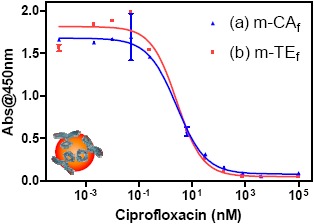
m-ELISA calibration curves of the different ELISA formats using the immunoreagents produced and the biomodified magnetic beads. (**a**) CA_f_, in blue; (**b**) TE_f_, in red. Each point was the average of at least three-well replicates, and the assays were run on two different days.

The main advantages of the use of magnetic beads in electrochemical immunosensors are their easy manipulation, the increase in the surface area, faster assay kinetics, improved washing and separation steps, the possibility of renewing the surface of the electrode and perspectives for system automation and miniaturization [11,12]. In this sense, two types of biomodified magnetic beads were prepared using tosyl-activated magnetic beads, ones by covalent binding of the Ab171 and others by covalent binding of the coating antigen 11-BSA. The biomodified magnetic beads were characterized by m-ELISA and both types of beads show a very good performance in m-ELISA, keeping the detectability that the same immunoreagents showed in ELISA (see Figure 3 and Table 2).

The yield of the biomodification was checked by testing the protein content from the supernatant before and after the bioconjugation using UV at 280 nm, by interpolation the value of supernatant into a calibration line built using the corresponding protein (Rabbit IgG and BSA). In both cases was higher than 80%.

As in the case of ELISA, the m-ELISA parameters found were similar for both formats. Since production of 11-HRP was more cost and time effective, the TE_f_ was selected to continue with the development of the AIMS, using the Ab171-MB.

### 3.3. Specificity Assay

In order to prove that the generic pattern of fluoroquinolone recognition was maintained in the magneto amperometric immunosensor, the specifitity of the new immunoreagents was tested using the TE_f_. Calibration curves for fluoroquinolones regulated by the UE were built in PBST_Ca_ buffer and were run using ELISA and m-ELISA. For both assays, the standard curves for all fluoroquinolones were fitted to the four-parameter equation mentioned above with very good correlation coefficients (≥0.985). Table 3 shows the values of IC_50_, cross reactivity percentage, with respect to the ciprofloxacin and the MRLs values, fixed by UE for milk. Detectability were very similar for both assays and close to the indirect ELISA previously reported [17], with a slight increase of IC_50_ for m-ELISA respect to the microplate-based ELISA except for the oxolinic acid. In all cases the IC_50_ values were lower than 10 μg/L and seven fluoroquinolones congeners were recognized at concentrations below to the MRLs established by the UE in milk.

### 3.4. Milk Matrix Effect

The term matrix effect is defined as the combined effect of all components of the sample other than the analyte on the measurement of the quantity [25]. Milk is a complex matrix composed by thousands of compounds, including high contents of fat, protein, carbohydrates, and minerals [26], which can affect the antibody recognition event. For this reason, the matrix effect was evaluated in whole milk free of fluoroquinolones by preparing standard curves of ciprofloxacin in milk, and milk diluted several times in PBST_Ca_ buffer, and running them in ELISA and m-ELISA. The calibration curves obtained under such conditions were compared to that built in just PBST_Ca_ buffer.

**Figure 4.**
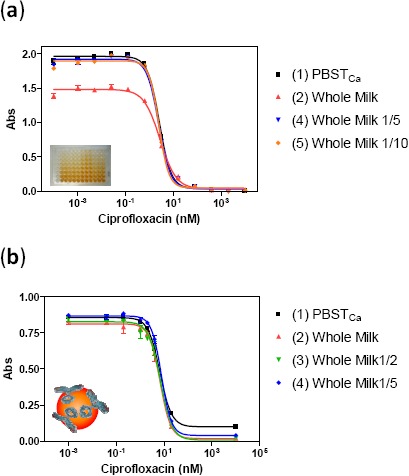
Milk matrix effect using (**a**) ELISA and (**b**) m-ELISA for the TE_f_. Curves were built in (1) PBST_Ca_ buffer, (2) Whole milk, (3) Whole milk diluted two times in PBST_Ca_ buffer, (4) Whole milk diluted five times in PBST_Ca_ buffer and (5) Whole milk diluted 10 times in PBST_Ca_ buffer. Each point was the average of at least three-well replicates, and the assays were run on two different days.

In ELISA assay, the matrix effect observed consisted in a decrease of the 25% of the maximum absorbance when the calibration curve was built directly in whole milk, while the calibration curves built in milk diluted more than five times in buffer were parallel to the one built in buffer (see Figure 4). Despite the decrease in maximum absorbance, the IC_50_ remains constant with values of 0.87 μg/L for buffer and 0.92 μg/L for milk. Regarding m-ELISA assay, no matrix effect was observed, even in raw milk without any dilution. The calibration curves showed a perfect parallelism to the buffer curve (see Figure 4). The fact that the matrix effect was fully eliminated in m-ELISA reveals the advantages of using magnetic particles in the assay and suggests the possibility of directly analyzing whole milk samples with m-ELISA format without any kind of sample pretreatment, allowing us to keep a very good detectability.

### 3.5. Amperometric Magneto Immunosensor

**Figure 5.**
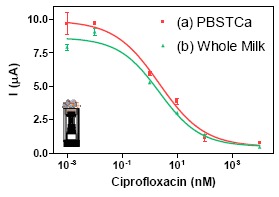
Calibration curves for ciprofloxacin with electrochemical readout using AMIS. (**a**) In PBST_Ca_ in red (**b**) In whole milk in green. Each point was the average of at least three-well replicates, and the assays were run on two different days.

Electrochemical readout for the implementation of the AMIS was addressed once the reagents (enzyme tracer and modified magnetic beads) were fully characterized by ELISA and m-ELISA. From two-dimensional experiments, optimum concentrations of Ab171-MB (0.25 mg/mL) and 11-HRP (0.5 μg/mL) were adjusted to obtain a final current of 7–10 μA after 30 min of immunochemical reaction in absence of analyte. Standard solutions of ciprofloxacin (from 0.01 to 10,000 nM in PBST_Ca_ buffer) were mixed with the biomodified magnetic beads Ab171-MB and 11-HRP (see experimental Section 2.9), incubated for 30 min and, captured with the m-GEC. Measurement of the immunocomplexes formed was performed in the electrochemical cell. The detectability of the AMIS in buffer, in term of its IC_50_ recorded was 0.74 μg/L, while the LOD, in term of the IC_80_, was 0.02 μg/L (see Table 4). The same experiment was performed preparing the same standard solutions in undiluted whole milk to check if the matrix affected the electrochemical readout, obtaining and IC_50_ of 0.74 μg/L, while the LOD was 0.009 μg/L (see Table 4), which indicated that the results obtained by the m-ELISA could be reproduced also with the immunosensor. Figure 5 shows the curves generated, in buffer and in milk. demonstrating the absence of significant non specific interferences caused by the milk in the AMIS, as in the case of m-ELISA. Both calibration curves, the one prepared in buffer and the one prepared in milk are almost identical, opening the possibility to prepare the calibration curves in buffer. Moreover, since the LOD is much below of the MRL values established by the EC, the milk will have to be diluted with buffer in order to use the present immunosensor for screening purposes, reducing even more the probability to produce non specific interferences in the assay.

Comparing the parameters obtained in m-ELISA and in AMIS, IC_50_ and LOD are quite similar, only the slope of the curve is lower in the case of AMIS. Thus, we can conclude that using the AMIS developed it is possible to analyze directly fluoroquinolone residues milk samples without any sample treatment.

On the other hand, several samples can be simultaneously processed with the aid of magnetic racks. In this work, up to 24 samples have been measured at the same time in 4.5 h.

## 4. Conclusions

An electrochemical magneto-immunosensor for the detection of fluoroquinolone antibiotics in milk has been developed. Time of the analysis has been decreased at half by the use of two new enzyme labeled immunoreagents, prepared by covalent binding of antibody and hapten for fluoroquinolones to the enzyme HRP using cyanuric chloride as crosslinker. In addition, magnetic beads have been biofunctionalized using fluoroquinonole specific antibodies and antigen bioconjugates. All immunoreagents, including magnetic beads, have been characterized by ELISA and m-ELISA using two formats, ET_f_ and CA_f_. The parameters extracted from this characterization indicate that detectability is not dependent on the format or type of assay. Studies of selectivity indicate that the immunoreagents developed are able to detect a significant number of the fluoroquinolone congeners regulated by UE with values of IC_50_ below 10 μg/L. The use of magnetic beads completely eliminates the matrix effect caused by milk due to improvement washing steps and better kinetic profile during the immunochemical reaction. Samples of milk can be directly analyzed without any sample treatment or dilution using AMIS approach and also using m-ELISA for high-throughput screening purposes, both of them with excellent detection limits (LOD for ciprofloxacin 0.009 μg/L for AMIS and 0.034 μg/L for m-ELISA). The simplicity of the electrochemical magneto immunosensor presented here makes it suitable for rapid and cheap semiquantitative and quantitative on-site analysis of fluoroquinolones in milk by nonqualified personnel.

## Figures and Tables

**Table 1.** Features of the different ELISA formats using the immunoreagents produced.

	CA_f_ (*11-BSA/Ab171-HCH*)	TE_f_ (*As171/11-HCH*)
Amax	0.600	2.318
Amin	0.026	0.023
IC_50_ (μg/L)	0.84 ± 0.39	0.86 ± 0.34
Slope	−0.808	−1.436
Dynamic range (μg/L)	0.16–2.20	0.33–2.35
LOD (μg/L)	0.060	0.086
R^2^	0.9854	0.9945

**Table 2.** Features of the different m-ELISA formats using the immunoreagents produced and the biomodified magnetic beads.

	m-CA_f_ (*11-BSA-MB/Ab171-HCH*)	m-TE_f_ (*Ab171-MB/11-HCH*)
Amax	1.679	1.814
Amin	0.081	0.052
IC_50_ (μg/L)	0.88 ± 0.42	0.89 ± 0.41
Slope	−0.799	−0.901
Dynamic range (μg/L)	0.15–5.47	0.10–3.73
LOD (μg/L)	0.052	0.034
R^2^	0.984	0.985

**Table 3.** Values of IC_50_ and cross-reactivity percentage of related fluoroquinolone compounds in the ELISA and m-ELISA.

	ELISA		m-ELISA		MRL [24]
		
	IC_50_ [μg/L]	%CR	IC_50_ [μg/L]	%CR	In Milk [μg/L]
Ciprofloxacin	0.93	100	1.31	100	100
Enrofloxacin	1.23	82	2.04	66	100
Danofloxacin	7.34	14	12.5	11	30
Difloxacin	1.42	86	2.34	70	Banned
Marbofloxacin	2.56	41	8.93	16	75
Flumequine	1.94	39	4.82	22	50
Oxolinic Acid	7.01	11	4.30	24	Banned

**Table 4.** Features of the amperometric magneto-immunosensor (AMIS) in PBST_CA_ buffer and whole milk samples using ciprofloxacin as standard.

	PBST_Ca_	Whole Milk
Imax (μA)	9.916	8.641
Imin (μA)	0.472	0.492
IC_50_ (μg/L)	0.74 ± 0.50	0.74 ± 0.48
Slope	−0.532	−0.586
Dynamic range (μg/L)	0.063–8.05	0.043–7.38
LOD (μg/L)	0.017	0.009
R^2^	0.980	0.981
